# Integrated Management Strategies for Diabetes Mellitus and Hypertension: A Systematic Review

**DOI:** 10.7759/cureus.103893

**Published:** 2026-02-19

**Authors:** Amit Nampalliwar, Prashant Uttam Sasane, Devendra Singh, Santosh Kumar Sahu, Sheetal Surykant Chavan, Prashant Nareshrao Deshmukh

**Affiliations:** 1 Department of Roga Nidan and Vikriti Vigyana (Pathology), Government Ayurved College and Hospital, Bilaspur, IND; 2 Department of Roga Nidan and Vikriti Vigyana (Pathology), Pt. Deendayal Upadhyay Memorial Health Science and Ayush University of Chhattisgarh, Raipur, IND; 3 Department of Kayachikitsa (Medicine), All India Institute of Ayurveda, Goa, IND; 4 Department of Roga Nidan and Vikriti Vigyana (Pathology), Mahayogi Guru Gorakhnath Ayush University, Gorakhpur, IND; 5 Department of Roga Nidan and Vikriti Vigyana (Pathology), Prakash Institute of Ayurvedic Medical Sciences and Research, Bulandshahr, IND; 6 Department of Shalakya Tantra (Ophthalmology), Government Ayurved College and Hospital, Bilaspur, IND; 7 Department of Roga Nidan and Vikriti Vigyana (Pathology), Ayurveda Seva Sangh Ayurveda Medical College, Nashik, IND; 8 Department of Roga Nidan and Vikriti Vigyana (Pathology), National Institute of Ayurveda, Deemed-to-be-University, Jaipur, IND

**Keywords:** diabetes mellitus, evidence synthesis, hypertension, integrated care, multidisciplinary management

## Abstract

Diabetes mellitus (DM) and hypertension frequently coexist and together substantially increase the risk of cardiovascular morbidity, mortality, and healthcare burden worldwide. Despite growing recognition of their shared pathophysiology, clinical management often remains fragmented, creating uncertainty regarding the optimal integration of care strategies. This systematic review aimed to synthesise current evidence on integrated management approaches for adults with coexisting DM and hypertension, with emphasis on intervention models and clinical outcomes. A systematic literature search was conducted in PubMed, Scopus, and Web of Science for studies published between 2015 and 2025. Eligible studies included randomised controlled trials and comparative observational studies evaluating integrated or combined management strategies. Eleven studies met the inclusion criteria and were synthesised systematically due to heterogeneity in study designs and outcome reporting. Integrated interventions commonly combined pharmacological optimisation, lifestyle modification, multidisciplinary team care, and technology-assisted support. Reported outcomes included improvements in glycaemic control, blood pressure regulation, cardiometabolic risk factors, and care process indicators. The findings highlight the consistent application of coordinated care frameworks across diverse healthcare settings, reflecting a shift toward comprehensive cardiometabolic risk management. Integrated strategies demonstrate potential to enhance care coordination, patient engagement, and alignment with patient-centred care models. The evidence supports integrated management as a viable and clinically relevant approach for addressing the complex needs of individuals with coexisting DM and hypertension, reinforcing the importance of coordinated strategies in contemporary chronic disease management.

## Introduction and background

Diabetes mellitus (DM) and hypertension are two of the most prevalent non-communicable diseases in the world, causing a substantial proportion of global cardiovascular morbidity and mortality [1]. Their steadily increasing prevalence is directly related to the ageing of the population, urbanisation, sedentary lifestyle, and rising obesity rates, particularly among low- and middle-income countries [2]. Epidemiological trends consistently demonstrate that DM and hypertension frequently coexist, and it has been estimated that more than half of all patients with type 2 diabetes experience high blood pressure at some point during their illness [3]. The combination of DM and hypertension confers a considerably greater risk of both macrovascular and microvascular complications compared with either condition alone [4]. Hyperglycaemia and hypertension interact to increase endothelial dysfunction, vascular inflammation, and arterial stiffness, which contribute to atherosclerosis and target organ damage [5]. These pathophysiological interactions increase the incidence of ischemic heart disease, stroke, chronic kidney disease, and heart failure among affected individuals [6].

The relationship between DM and hypertension has solid biological foundations, encompassing insulin resistance, activation of the renin-angiotensin-aldosterone system, oxidative stress, and low-grade systemic inflammation [7]. These mechanisms not only promote disease development but also complicate clinical management, as intervention in one pathway may influence the other. Patients with both conditions typically require complex treatment plans and long-term surveillance to maintain risk factors within recommended clinical targets [8]. Clinical management has traditionally followed disease-specific pathways and speciality-based care models [9]. Although this approach has led to significant therapeutic advances, it has also created gaps in care delivery and inconsistent treatment outcomes. Limited coordination among providers, inconsistent follow-up, and low patient engagement may contribute to suboptimal blood pressure and glycaemic control in routine practice [10].

In response to these challenges, integrated management approaches have emerged as strategies designed to address the multidimensional needs of patients with coexisting DM and hypertension [11]. These approaches emphasise simultaneous management of multiple cardiometabolic risk factors through a combination of pharmacological therapy, lifestyle modification, and structured healthcare delivery. Multifactorial intervention studies demonstrate that comprehensive risk factor control can significantly reduce cardiovascular events and diabetes-related complications [12].

Lifestyle-oriented interventions

Lifestyle modification is a central component of comprehensive management, with evidence supporting improvements in haemodynamic and metabolic outcomes [13]. Nutritional modification, regular physical activity, weight reduction, and smoking cessation have been shown to lower blood pressure, enhance insulin sensitivity, and reduce systemic inflammation. These measures complement pharmacological treatment and contribute to improved disease control and quality of life [14].

Multidisciplinary and structured care models

Integrated care frequently involves coordinated input from multidisciplinary teams, structured follow-up systems, and shared clinical targets. Such models aim to improve communication among healthcare providers, enhance continuity of care, and promote patient-centred decision-making.

Digital health and technology-supported interventions

Digital health tools, including telemonitoring, electronic decision-support systems, and remote consultations, have increasingly been incorporated into integrated management frameworks to support monitoring, adherence, and timely treatment adjustments.

Despite the growing evidence supporting integrated care models, implementation across healthcare systems remains uneven. Differences in resources, infrastructure, and professional training limit consistent adoption. Existing research evaluating integrated strategies is heterogeneous in study design, intervention components, and outcome measures, which constrains comparability and limits evidence synthesis. A clearer understanding of the clinical impact of integrated management interventions on patients with DM and hypertension is therefore required. Synthesising current evidence can identify effective components, inform clinical decision-making, and support the development of patient-centred care models. This need is amplified by the rising global burden of cardiometabolic disease and the demand for sustainable healthcare delivery models.

An increasing focus has been placed on combined management of diabetes and hypertension in response to recognition of the complex needs of patients with coexisting cardiometabolic conditions. Scientific evaluation of these strategies requires consideration of clinical outcomes, intervention components, and care delivery structures across diverse study designs. Integrating findings from multiple study types may clarify the contribution of coordinated strategies to long-term health outcomes and support more systematic, patient-centred approaches to managing these common comorbidities.

Objectives of the review

The objective of this systematic review is to evaluate the effectiveness of integrated management strategies in patients with coexisting DM and hypertension. The review synthesises evidence on combined pharmacological, lifestyle, and care delivery interventions and their impact on glycaemic control, blood pressure, and cardiovascular outcomes. It also seeks to identify key components of integrated care models associated with improved clinical outcomes and to inform future clinical practice and research.

## Review

Methodology

Search Strategy

A systematic literature search was conducted across multiple electronic databases to identify studies evaluating integrated management strategies for DM and hypertension. The databases searched included PubMed, Scopus, and Web of Science. The search strategy combined controlled vocabulary terms and free-text keywords related to DM, hypertension, integrated care, combined management, and multidisciplinary interventions. Boolean operators were applied to refine search outputs. Only studies involving human participants, published in English, and within the time frame of 2015 to 2025 were considered. Duplicate records were removed before screening. Reference lists of included articles were manually reviewed to identify additional relevant studies.

Controlled vocabulary terms and free-text keywords were combined using Boolean operators (AND/OR) with database-specific syntax to optimise retrieval. Core search concepts included diabetes mellitus, hypertension, and integrated or multidisciplinary care approaches. In PubMed, Medical Subject Headings (MeSH) were used alongside free-text terms to improve sensitivity. Key MeSH terms included “Diabetes Mellitus” and “Hypertension,” combined with terms related to integrated care models such as “Integrated Delivery of Health Care,” “Patient Care Team,” and “Telemedicine.”

This systematic review was conducted and reported in accordance with the Preferred Reporting Items for Systematic Reviews and Meta-Analyses (PRISMA) guidelines.

Study Selection

Titles and abstracts retrieved from the database search were screened for relevance. Full texts of potentially eligible studies were then assessed against predefined eligibility criteria. Studies not meeting the inclusion criteria at any stage were excluded.

Eligibility Criteria

Inclusion criteria: Eligible studies included adult populations diagnosed with both DM and hypertension and evaluated integrated or combined management strategies. Randomised controlled trials, cohort studies, and comparative observational studies were included if they reported at least one of the following outcomes: glycaemic control, blood pressure outcomes, or cardiovascular-related complications. Studies conducted in primary or secondary care settings were considered.

Exclusion criteria: Studies focusing on a single condition without integrated management, editorials, case reports, conference abstracts, non-English publications, paediatric populations, and studies lacking adequate outcome data were excluded.

Data Extraction

Data were extracted using a standardised data extraction framework. Extracted variables included study design, sample size, participant characteristics, intervention components, duration of follow-up, comparator groups, and reported clinical outcomes. Outcomes of interest comprised measures of glycaemic control, blood pressure regulation, cardiovascular outcomes, and care-related indicators.

Data Synthesis

A quantitative meta-analysis was not performed due to clinical and methodological heterogeneity across included studies. Differences were observed in study design, intervention components, outcome definitions, and duration of follow-up. These variations reduced comparability across studies and limited the suitability of statistical pooling.

Findings were synthesised narratively and presented in structured tables to facilitate comparison. Outcomes were grouped into glycaemic outcomes, blood pressure outcomes, cardiovascular outcomes, and healthcare-related measures. Study-specific statistical results, including effect estimates and significance levels where reported, were summarised descriptively. Formal statistical heterogeneity measures were not calculated, as pooled quantitative analysis was not undertaken.

Quality Assessment

Methodological quality of included studies was assessed to evaluate the strength and reliability of evidence. Studies were appraised based on clarity of objectives, appropriateness of design, comparability of intervention groups, validity of outcome measures, and adequacy of follow-up duration. Randomised controlled trials and observational studies were evaluated using design-appropriate methodological principles. Study quality was considered during the interpretation of findings, with greater weight assigned to studies demonstrating more rigorous methodology.

Risk-of-Bias Assessment

Risk of bias was evaluated at the study level to identify potential sources of systematic error. The Cochrane Risk-of-Bias tool (ROB 2) was applied to randomised controlled trials, assessing bias related to the randomisation process, deviations from intended interventions, missing outcome data, outcome measurement, and selective reporting. Each domain was judged according to ROB 2 guidance, and an overall risk-of-bias rating was assigned to each randomised study.

Results

Study Selection

**Figure 1:**
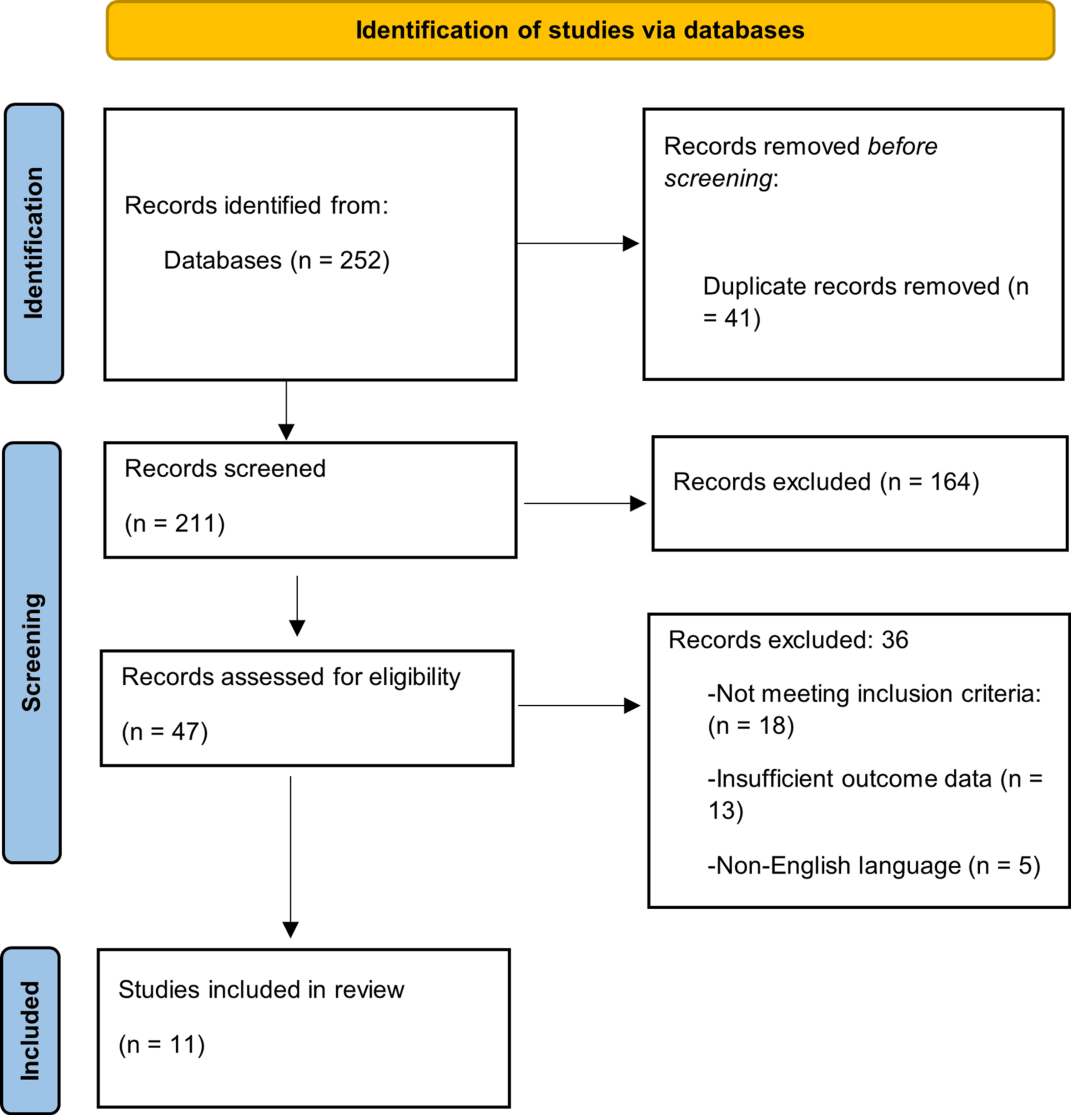
PRISMA flow diagram PRISMA: Preferred Reporting Items for Systematic Reviews and Meta-Analyses

The electronic database search identified 252 records. Duplicate removal eliminated 41 records, leaving 211 unique records for title and abstract screening. Screening led to the exclusion of 164 records that did not meet the scope of the review. Full-text assessment was conducted for 47 articles to determine eligibility. This assessment resulted in the exclusion of 36 articles because they did not meet the inclusion criteria (n = 18), provided insufficient or unclear outcome data (n = 13), or were published in a non-English language (n = 5). A total of 11 studies met all eligibility requirements and were included in the final review. Figure 1 presents the PRISMA flowchart illustrating the study selection process and the reasons for exclusion at each stage.

Characteristics of Included Studies

The studies included were randomised controlled trials, observational comparative studies and were organised in different geographic areas. Adult patients who had hypertension as well as DM were included in the study population. The sample sizes used in the studies were quite different, and the follow-up period was a short-term intervention as well as a program lasting several months or more. The interventions were offered in the primary care environment, outpatient clinics and community-based settings. Comparators in general were given normal care or normal management of the disease. Table 1 shows the top features and comparative results of the concerned studies.

**Table 1:** Overview of integrated management studies

Study	Intervention type	Study design and population	Main outcomes assessed	Key findings
Cheung et al. [15]	Mobile health self-management support	Pragmatic randomised controlled trial; adults with diabetes and/or coronary heart disease	Blood pressure control	Text-messaging support was associated with improved blood pressure outcomes compared with usual care.
Mihevc et al. [16]	mHealth home telemonitoring	Multicenter randomised controlled trial; older adults with hypertension and type 2 diabetes.	Blood pressure, glycaemic control	Telemonitoring demonstrated improvements in blood pressure and glycaemic parameters over 12 months.
Kindlovits et al. [17]	Exercise and dietary intervention	Interventional study; elderly patients with type 2 diabetes and hypertension	Blood pressure, metabolic outcomes	Combined hypoxic exercise and dietary modification showed favourable blood pressure responses.
Vlacho et al. [18]	Multicomponent healthcare intervention	Controlled intervention study; adults with poorly controlled type 2 diabetes	Blood pressure, lipid profile	Integrated intervention improved blood pressure and lipid outcomes
Trento et al. [19]	Self-management education	Randomised controlled trial; adults with type 2 diabetes	Blood pressure	Structured education was associated with improved blood pressure control
Morelli et al. [20]	Digital health–based diabetes program	Quasi-experimental study; adults with type 2 diabetes	Quality of care, metabolic outcomes	Digital health intervention improved care quality indicators
Siaw et al. [21]	Pharmacist-involved collaborative care	Randomised controlled trial; high-risk patients with type 2 diabetes	Clinical and cost outcomes	Collaborative care improved clinical outcomes and cost-effectiveness
Wu et al. [22]	Interdisciplinary collaborative care with family empowerment	Controlled clinical study; patients with comorbid diabetes and hypertension	Blood pressure, glycaemic control	The integrated care model improved blood pressure and glucose control
Pence et al. [23]	Integrated chronic disease care	Cluster-randomised implementation trial; adults with diabetes and hypertension	Care integration outcomes	Integrated screening and treatment strategies were successfully implemented.
Kivuyo et al. [24]	Integrated management of multiple chronic conditions	Pragmatic cluster-randomised trial; adults in sub-Saharan Africa	Blood pressure, glycaemic outcomes	Integrated care improved the management of diabetes and hypertension
Oh et al. [25]	Mobile health integrative intervention	Crossover study: adults with diabetes and hypertension	Blood pressure, glycaemic outcomes	Mobile health intervention demonstrated improvements in cardiometabolic outcomes.

Integrated Management Interventions

Among the integrated management strategies reported across the included studies, interventions were broadly grouped into multidisciplinary care models, technology-assisted interventions, lifestyle-based programmes, and coordinated pharmacological management. Multidisciplinary care models typically involve coordinated management delivered by a team comprising physicians, nurses, pharmacists, and allied health professionals. Technology-assisted interventions included mobile health applications, telemonitoring, and digital platforms designed to support combined diabetes and hypertension monitoring and follow-up. Lifestyle-based programmes focused on structured dietary counselling, physical activity guidance, and behaviour change support. Coordinated pharmacological management emphasised integrated prescribing, titration, and monitoring of antihypertensive and antidiabetic therapies. The included studies varied substantially in the composition, intensity, and duration of interventions. Table 2 summarises the integrated care models and implementation characteristics across studies.

**Table 2:** Models of integrated care and implementation characteristics

Integrated care model	Key components	Healthcare setting	Target population	Reference
Mobile health–supported self-management	Text messaging, patient education	Outpatient care	Adults with diabetes and hypertension	[15]
Telemonitoring-based integrated care	Remote monitoring, clinician feedback	Home-based care	Older adults with type 2 diabetes and hypertension	[16]
Lifestyle-integrated intervention	Exercise and dietary modification	Clinical setting	Elderly patients with cardiometabolic disease	[17]
Multidisciplinary healthcare model	Physician-led team care	Primary care	Adults with poorly controlled diabetes	[18]
Pharmacist-involved collaborative care	Medication optimisation, follow-up	Outpatient clinic	High-risk patients with diabetes	[21]
Integrated chronic disease clinic	Coordinated screening and treatment	Community-based setting	Adults with diabetes and hypertension	[23]
Digital health integrative model	App-based monitoring, education	Ambulatory care	Adults with cardiometabolic conditions	[25]

**Figure 2:**
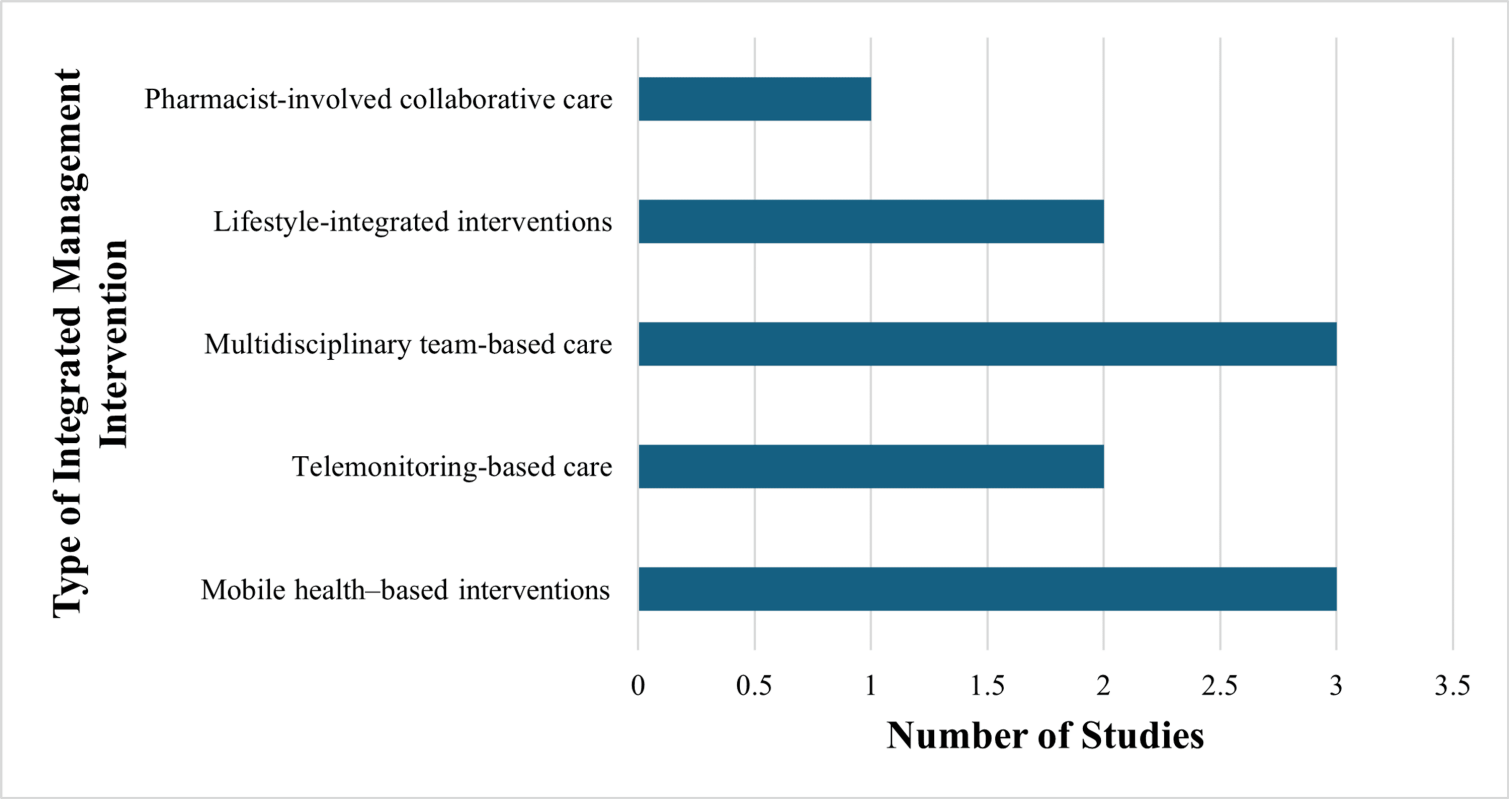
Distribution of integrated management intervention types

Figure 2 illustrates the distribution of integrated management intervention types across included studies.

Clinical Outcomes

Clinical outcomes reported across included studies primarily included glycaemic control, measured using fasting blood glucose and glycated haemoglobin, and blood pressure outcomes, reported as systolic and diastolic blood pressure levels. Additional cardiometabolic outcomes were reported in several studies, including lipid profiles, renal function parameters, and body weight. Some studies also reported broader outcomes such as cardiovascular events, hospitalisation rates, medication adherence, and healthcare utilisation. The included studies varied in outcome definitions, measurement methods, and follow-up time points, which limited direct comparability across studies. Table 3 summarises the clinical outcomes reported in relation to integrated management strategies and presents the direction of reported outcome changes across intervention models.

**Table 3:** Clinical outcomes associated with integrated management strategies HbA1c: Glycated haemoglobin, BP: Blood pressure

Outcome domain	Type of integrated intervention	Outcome measure(s) reported	Outcome reported	Reference
Glycaemic control	Technology-assisted integrated care	HbA1c	HbA1c reduction	[16]
Blood pressure control	Mobile health self-management	Systolic and diastolic BP	Reduction in SBP and DBP	[15]
Cardiometabolic risk	Multicomponent healthcare intervention	Lipid profile, BP	Improvement in lipid profile and BP outcomes	[18]
Blood pressure control	Self-management education	Systolic BP	Reduction in systolic BP	[19]
Glycaemic and BP control	Interdisciplinary collaborative care	Fasting glucose, BP	Improvement in fasting glucose and BP outcomes	[22]
Cardiovascular indicators	Integrated chronic disease care	BP and metabolic parameters	Improvement in BP and metabolic parameters	[24]
Glycaemic and BP outcomes	Mobile health integrative intervention	HbA1c and BP	Improvement in HbA1c and BP outcomes	[25]

**Figure 3:**
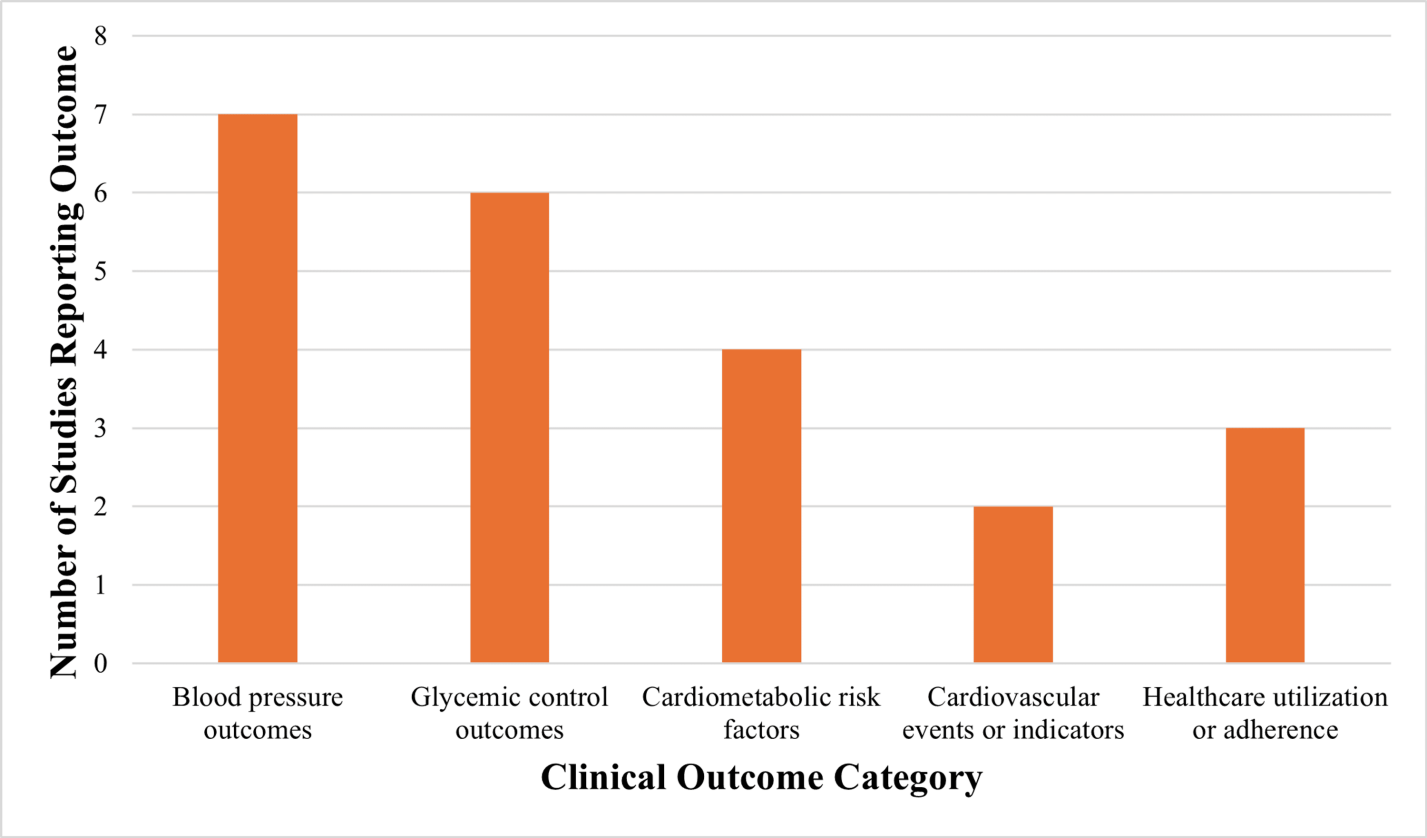
Clinical outcomes reported across included studies

Figure 3 depicts the frequency of reported clinical outcomes among the included studies.

Quality of Included Studies

The methodological quality of the included studies varied. In randomised controlled trials, intervention protocols and outcome measures were generally well-defined. In observational studies, greater variability was noted in group comparability and outcome assessment. Differences were also observed in follow-up duration and completeness of outcome reporting. Study quality ratings are summarised descriptively, and key appraisal findings are reflected in the interpretation of results.

Risk-of-Bias Assessment

The included studies demonstrated varying levels of risk of bias. Randomised controlled trials showed differences in bias related to randomisation procedures, outcome measurement, and reporting. Observational studies showed greater inconsistency in baseline comparability, control of confounding, and outcome assessment. Table 4 summarises the overall risk of bias judgments across included studies.

**Table 4:** Risk-of-bias assessment of included studies

Study	Study design	Selection bias	Performance bias	Detection bias	Overall risk of bias
Cheung et al. [15]	Randomised controlled trial	Low	Low	Low	Low
Mihevc et al. [16]	Randomised controlled trial	Low	Low	Low	Low
Kindlovits et al. [17]	Interventional study	Moderate	Moderate	Moderate	Moderate
Vlacho et al. [18]	Controlled intervention study	Moderate	Moderate	Low	Moderate
Trento et al. [19]	Randomised controlled trial	Low	Moderate	Low	Moderate
Morelli et al. [20]	Quasi-experimental study	Moderate	Moderate	Moderate	Moderate
Siaw et al. [21]	Randomised controlled trial	Low	Low	Low	Low
Wu et al. [22]	Controlled clinical study	Moderate	Moderate	Moderate	Moderate
Pence et al. [23]	Cluster-randomised trial	Low	Moderate	Low	Moderate
Kivuyo et al. [24]	Cluster-randomised trial	Low	Moderate	Low	Moderate
Oh et al. [25]	Crossover study	Moderate	Moderate	Low	Moderate

Discussion

This systematic review examined studies on integrated management strategies in people with coexisting DM and hypertension, focusing on the composition of interventions, reported outcomes, and care models. The results indicate that integrated strategies consistently aim to address metabolic and cardiovascular risk factors simultaneously through coordinated pharmacological management, lifestyle interventions, and multidisciplinary care delivery. These approaches reflect a broader shift across healthcare settings away from fragmented disease-specific management and toward integrated, patient-centred models that recognise the interdependence of cardiometabolic conditions.

DM and hypertension represent significant clinical challenges because they share overlapping aetiology and jointly amplify cardiovascular risk [26]. Epidemiological studies have shown a higher prevalence of atherosclerotic disease, renal impairment, and cardiovascular mortality in individuals with both conditions compared with those affected by either condition alone [1]. Integrated management strategies address these combined risks by targeting hyperglycaemia, elevated blood pressure, and related metabolic disturbances within a unified therapeutic framework [2].

Multifactorial and Risk-Factor-Based Approaches

Multifactorial intervention strategies have been associated with improved cardiometabolic outcomes due to their emphasis on managing multiple risk factors concurrently [27]. Prior studies indicate that effective control of blood glucose, blood pressure, and lipid levels reduces microvascular and macrovascular complications in individuals with type 2 diabetes [3]. These findings support integrated care models that prioritise holistic risk-factor management rather than isolated disease-specific treatment [4].

Lifestyle-Based Components

Lifestyle modification is a key element of integrated management strategies because of its broad physiological benefits and applicability across patient groups [5]. Dietary interventions, physical activity, and weight management have been shown to improve insulin sensitivity and reduce blood pressure [28]. Incorporation of these measures into structured care programmes supports sustained behavioural change and complements pharmacological therapy [6]. This integration reflects a comprehensive approach to chronic cardiometabolic disease management [7].

Multidisciplinary Care Models

The use of multidisciplinary healthcare teams has become increasingly important in the management of complex chronic diseases [29]. Collaborative care models involving physicians, nurses, pharmacists, and other health professionals may improve continuity of care and coordinated decision-making [8]. These models are particularly relevant for individuals with DM and hypertension who require complex medication regimens and regular monitoring [9]. Multidisciplinary integration may also improve patient engagement and adherence to treatment plans [10].

Digital Health and Technology-Supported Interventions

Digital health technologies have emerged as important tools within integrated management models by enabling remote monitoring, self-management support, and patient education [11]. Technology-assisted interventions can enhance communication between patients and healthcare providers and support timely adjustments in treatment plans. Integration of digital tools within care delivery aligns with broader movements toward patient-centred and evidence-based healthcare systems [12].

Patient-Centred and System-Level Implications

Integrated management approaches align with patient-centred care principles by supporting individualised treatment based on patient needs and healthcare context [1]. Patients with coexisting DM and hypertension often experience high treatment burden, and coordinated care may reduce conflicting recommendations and duplication of services [2]. Integrated models may contribute to more consistent achievement of therapeutic targets and improved care experiences [3]. At the health system level, integrated strategies are aligned with improving efficiency and coordination across care levels [4]. Strengthening linkages between primary care, specialist services, and community-based resources may enhance continuity and equitable access to care [5]. Such approaches are particularly relevant in settings with high cardiometabolic disease burden and limited healthcare infrastructure [30].

The findings of this review reinforce the conceptual basis of integrated management of DM and hypertension, reflecting the interdependence of metabolic and cardiovascular pathways. The emphasis on combined interventions highlights the importance of addressing shared risk factors through integrated care planning [6]. The alignment of available evidence with integrated care delivery models underscores the relevance of pharmacological optimisation, lifestyle modification, and multidisciplinary coordination in routine clinical practice [14].

Limitations and Future Directions

There are a few limitations that one should consider while interpreting the findings of this review. The studies included were very heterogeneous in terms of design, intervention elements, period and outcome measures, which constrained direct comparability. The use of qualitative synthesis did not permit the quantitative estimation of the effect sizes. This heterogeneity also limited the feasibility of conducting a quantitative meta-analysis using a standardised framework. Reporting of variations and differences in follow-up periods could have contributed to the evaluation of outcomes. Moreover, the restriction to English language sources could have resulted in a lack of relevant evidence in non-English articles in various healthcare facilities and groups.

The standardised frameworks of intervention and outcome measures need to be given priority in future research to increase the comparability of studies. Future studies should adopt standardised intervention taxonomies and harmonised outcome reporting to enable quantitative synthesis and meta-analysis. Sustainable clinical impact should be studied in large-scale randomised trials with a long follow-up. Increased focus on implementation science could help in better understanding the role of the integrated care model in diverse health systems. Incorporating these findings into decision-making processes may strengthen policy development and practice, patient-reported outcomes and, of course, evaluation. Extended coverage of low-resource environments could also contribute towards informed usage of integrated management approaches in the world and also be sustainably applied.

## Conclusions

This systematic review synthesises current evidence on integrated management strategies for individuals with coexisting DM and hypertension, highlighting the growing emphasis on coordinated, patient-centred approaches to cardiometabolic care. The findings demonstrate that integrated models typically combine pharmacological optimisation, lifestyle modification, and multidisciplinary or technology-assisted care to address shared risk factors and complex treatment needs. Such approaches reflect recognition of the interconnected pathophysiology underlying diabetes and hypertension and the limitations of fragmented, disease-specific management. By focusing on concurrent control of glycaemic and blood pressure parameters, integrated strategies aim to improve clinical outcomes while enhancing care coordination and efficiency. The review highlights the diversity of intervention models employed across healthcare settings, demonstrating flexibility in implementation while upholding the core principles of comprehensive risk management. The evidence supports integrated management as a viable framework for addressing the substantial burden associated with coexisting diabetes and hypertension. The adoption of coordinated care strategies may facilitate the achievement of therapeutic targets, improve patient engagement, and align clinical practice with evolving models of chronic disease management. These conclusions reinforce the relevance of integrated approaches in advancing effective cardiometabolic care. Future research should prioritise standardized intervention frameworks and harmonised outcome measures to strengthen comparability across studies and enable quantitative meta-analysis.
